# *Acholeplasma laidlawii* PG8 Culture Adapted to Unfavorable Growth Conditions Shows an Expressed Phytopathogenicity

**DOI:** 10.1100/tsw.2007.25

**Published:** 2007-01-10

**Authors:** Vladislav M. Chernov, Natalia E. Moukhametshina, Yurii V. Gogolev, Tatiana N. Nesterova, Maxim V. Trushin, Olga A. Chernova

**Affiliations:** Kazan Institute of Biochemistry and Biophysics, Russian Academy of Sciences, Kazan, Russia

**Keywords:** Acholeplasma laidlawii PG8, phytopathogenicity, ultramicroforms

## Abstract

Mycoplasmas are the smallest, self-replicating, prokaryotic organisms with avid biochemical potential and spreading in higher eukaryotes in nature. In this study, *Acholeplasma laidlawii* PG8 cells were cultivated on a deficient medium for 480 days resulting in a mycoplasma culture that was adapted *in vitro* to unfavorable growth conditions. Cells that survive this condition had decreased sizes (about 0.2 μm) and increased phytopathogenicity. This resulted in more frequent appearance of various morphological alterations when plants of vinca (*Vinca minor* L.) were infected by adapted mycoplasma cells. The increasing pathogenicity was accompanied by changes in genome expression in these adapted cells. Further studies are needed to explore the exact mechanisms that permit adaptation to unfavorable growth conditions and changes in phytopathogenic potential.

## INTRODUCTION

Mycoplasmas are the smallest, self-replicating, prokaryotic organisms. Despite restricted biochemical properties, these bacteria spread in various biotic communities and are able to persist in higher eukaryotes through their circulation in nature. It seems that the strong adaptability of these organisms resides in molecular genetic principles that permit them to adapt to unfavorable growth conditions (UGC). In our previous reports, we took some initial steps in exploring this adaptability[1,2], however, our knowledge of this mycoplasma potential is still in preliminary stages.

*Acholeplasma laidlawii,* a "ubiquitous" mycoplasma, is found in soil, compost, and wastewaters, as well as in tissues of humans, animals, and plants[3]. Previously, we showed that the adaptive reactions of *A. laidlawii* PG8 to UGC were connected with transformation of the vegetative forms of the mycoplasma cells into ultramicroforms (UMF) resistant to biotic and abiotic stress factors[1,2], a process that is called nanotransformation. Nanotransformation is not limited to mycoplasmas and has been shown in a number of bacteria. In some bacteria, adaptation to UGC was followed by distortions of pathogenicity. In our prior model of plant infection by mycoplasmas, we showed that *A. laidlawii* PG8 could cause phytomycoplasmosis[4,5]. In this study, we tried to show whether adaptation to UGC could affect the phytomycoplasmosis capability of *A. laidlawii* PG8 culture cells.

## MATERIALS AND METHODS

*Acholeplasma laidlawii* PG8, a reference strain, was obtained from the N.F. Gamalei Research Institute of Epidemiology and Microbiology (Moscow, Russia). Mycoplasma cells were cultivated for 2 days at 37°C on Edward's medium (1 l of this medium consisted of tryptic extract of bovine heart – 850 ml, serum of horse blood – 100 ml, fresh yeast extract (5%) – 40 ml, glucose [40% solution] – 5 ml, benzylpenicillin [1,000,000 IE/ml] – 5 ml) to obtain a culture unadapted to UGC. To obtain a mycoplasma culture adapted to UGC, glucose and yeast extracts were eliminated from Edward's medium; microorganisms were kept in this condition for 480 days as was previously described[1]. To detect the titer of the colony-forming units (CFU), semifluid and solid-state variants of the Edward's medium with addition of 0.5% and 1.2% agarose were used. Two-dimensional protein electrophoresis was performed as was described by us[2]. PCR analysis of DNA was done using specific AL16LF and A23LR primers for amplifying nucleotide sequences of *A. laidlawii* PG8 rRNA operons as was previously reported[2].

The phytopathogenicity of the mycoplasma culture cells adapted and unadapted to UGC was investigated on substantive stalks of vinca (*Vinca minor* L.), a specific indicator of phytomycoplasmosis[6]. Both control and experimental groups consisted of 20 plants with similar size and age of the root. Plant seedlings were infected with mycoplasmas on the 15$^{th}$ day after rooting. It was hypothesized that adapted culture of *A. laidlawii* PG8 has an increased phytopathogenic potential. To compare a level of its phytopathogenicity, we knowingly used a lesser amount of *A. laidlawii* PG8 cells adapted to UGCs. Our presupposition that a decreased number of cells may cause increased phytopathogenic alterations (in comparison with unadapted culture) was totally confirmed in our study (see results). We injected 10 µL of *A. laidlawii* PG8 culture ($10^5$ CFU of adapted culture and $10^7$ CFU of unadapted mycoplasma culture) by microsyringe into the root waist. In the control group, plants were injected with 10 µL of sterile nutrient medium. Control and experimental plants were monitored for 6–8 weeks, a time necessary for manifestation of phytomycoplasmosis. The alterations in plant characteristics were recorded. These alterations were previously described[6] and included plant delay in bine growth, chlorosis, necrosis, leaf marcescence, and abnormalities in bine development.

A Hitachi-110 transmission electron microscope (Hitachi, Japan) was used for analysis of the *in vitro* cultivating mycoplasma cells. Ultrathin sections were obtained using LKB-III ultramicrotome (Sweden). The material under study was fixed with glutaraldehyde (2.5%) prepared on a 0.1 *M* phosphate buffer (pH 7.2) for 12 h. Then, the material was dehydrated using an acetone series and postfixed in 0.1% $OsO_4$ with addition of 34 mg/ml of saccharose.

Numerical data are presented as mean ± standard deviation. A probability level of $p < 0.05$ was considered significant[7].

## RESULTS

The adapted *A. laidlawii* PG8 cells showed significant reduction in size compared to unadapted cells (Fig. 1). On the solid medium, the UMF formed specific microcolonies 50–300 µm in size. This is in contrast to typical mycoplasma cells that form "fried eggs" colonies. The UMF were able to revert into original vegetative forms of *A. laidlawii* PG8 cells when transferring to the full Edward's medium, showing an evident viability and resistance to stress factors.

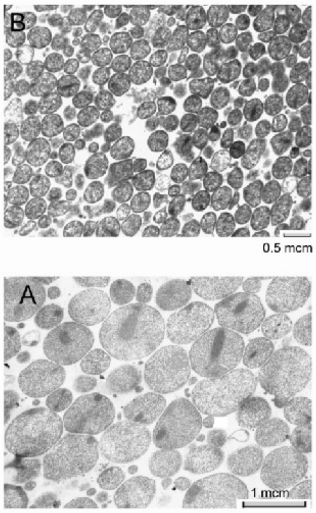

**FIGURE 1.** Electron micrography of *A. laidlawii* PG8 cells. Cultures were grown on Edward's medium for 2 days (A) and on the impoverished medium for 480 days (B).

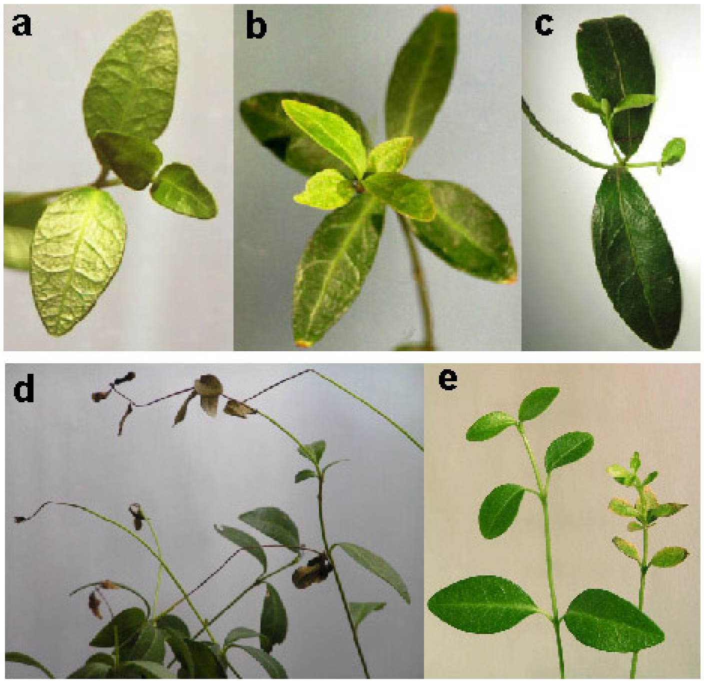

**FIGURE 2.** Morphosis in plants of *V. minor* L. infected by cells of *A. laidlawii* PG8 adapted culture. (a) Chlorosis and abnormalities of bine development (bulging of plant ribs); (b) necrosis (of the leaf edge); (c) abnormalities of bine development; (d) necrosis (of the apical leaves); (e) abnormalities of bine development (microplasia of the infected plant, right; normal plant, left).

Infecting plants of *V. minor* L. by *A. laidlawii* PG8 culture adapted *in vitro* to UGC resulted in the appearance of plant infection (Fig. 2). Plants infected by *A. laidlawii* PG8 culture unadapted to UGC showed significantly less signs of infection. For example, chlorosis was seen in 75% of plants infected with adapted mycoplasma in comparison with 40% in those with infection caused by unadapted mycoplasma. The other signs of infection were also more prevalent in adapted mycoplasma culture, such as necrosis by (50 vs. 25%), leaf marcescence (50 vs. 25%), and abnormalities of bine development (30 vs. 0%) (Table 1).

**TABLE 1**
**A Number of *V. minor* L. Plants with Various Morphological Deviations in Control and Experimental Groups***

| Variant of Experiment | Type of Phytomycoplasmosis | | | | Mean ± SD |
|---|---|---|---|---|---|
| | Chlorosis | Necrosis | Leaf Marcescence | Abnormalities of Bine Development | |
| Control | 3 (15) | 0 (0) | 0 (0) | 0 (0) | 0.75 ± 1.5 (3.75 ± 7.5%) |
| Unadapted culture | 8 (40) | 5 (25) | 5 (25) | 0 (0) | 4.50 ± 3.32 (22.5±16.58%) |
| Adapted culture | 15 (75) | 10 (50) | 10 (50) | 6 (30) | 10.25 ± 3.68 (51.25 ± 18.43%) |

* Absolute number of plants is indicated (percentage of the respective morphological deviations is shown in brackets).

The obtained results favor the hypothesis that adaptation *in vitro* of *A. laidlawii* PG8 culture to UGC was followed by a significant increase of its pathogenic potential.

## DISCUSSION

Previously, we showed that adaptation of *A. laidlawii* PG8 culture to UGC was followed by nanotransformation, transformation of the vegetative form of cells of the mycoplasma into UMF resistant to biotic and abiotic stress factors[1,2]. There were noticeable differences in the morphological, biochemical, and genetic molecular properties between the vegetative form of cells of the mycoplasma and its UMF.

As a result of the analysis of two-dimensional electrophoresis data, significant differences in the cell polypeptide spectra of *A. laidlawii* PG8 culture adapted to UGC and the unadapted one were registered (Fig. 3). This might be evidence for an essential reorganization of the mycoplasma genome expression during nanotransformation, entering the vegetative form of cells of the mycoplasma into UMF due to UGC.

Other researchers have shown that there is a reverse attenuation of PCR signals for DNA of bacterial cells during cultivation in UGC[8]. Appearance of differential amplification of *A. laidlawii* PG8 *rrn*A and *rrn*B nucleotide sequences due to dissociation of the cell culture population was reported in our recent study[9]. The data were considered to be useful to detect dissociation in cell population of the mycoplasma culture and to confirm transformation of the vegetative form of *A. laidlawii* PG8 cells into UMF in plant tissues.

In our previous experiments, we demonstrated that infecting by *A. laidlawii* PG8 culture unadapted to UGC was followed by the appearance of ultrastructural, morphophysiological, and biochemical deviations correlated with transformation of the vegetative form of the mycoplasma cells into UMF[1,2]. The data from our present study show that *A. laidlawii* PG8 culture adapted to UGC has higher expressive phytopathogenicity than the unadapted one. Even a 100-fold difference in a number of cells could not

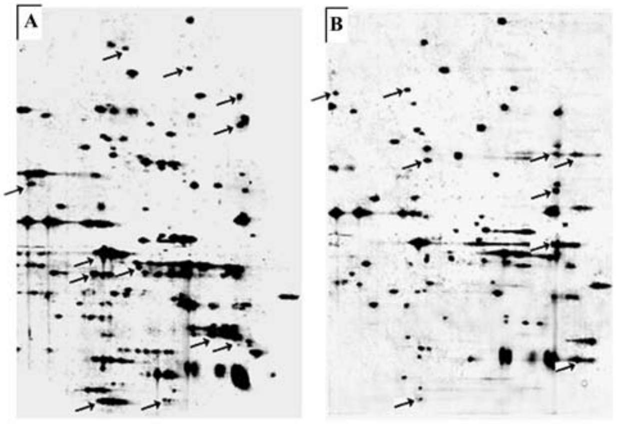

**FIGURE 3.** Electrophoregram of polypeptides of vegetative cells (A) and ultramicroforms (B) of *A. laidlawii* PG8. The arrows mark the polypeptides different in the mycoplasma cells.

influence the situation. In the plants infected by *A. laidlawii* PG8 unadapted to UGC culture, the symptoms of the mycoplasma infection were detected somehow later, and were less evident in comparison with the adapted mycoplasma culture (Fig. 4). A delayed manifestation of the mycoplasma infection by plants infected by unadapted mycoplasma could be related to the time that is needed for transformation of the vegetative form of cells into UMF. A comparison of the injected-adapted mycoplasma strain and the isolated one from the plants on biological differences remains to perform.

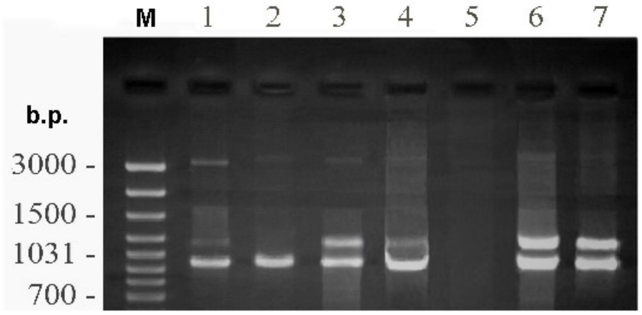

**FIGURE 4**. Detection of *A. laidlawii* PG8 by PCR with primers LA16LF and LA23LR in plants (*V. minor* L.). The lanes 1–4 contain PCR products of DNA extracted from plants infected with cells of adapted (1, 2) and unadapted (3, 4) culture of the mycoplasma through 4 days (1, 3) and 21 days (2, 4) after injecting. The lanes 5–7 correspond to negative (5) and positive (6, 7) controls – PCR products of DNA (230 ng) extracted from uninfected plants (5), mixed DNAs (230 ng/10 ng) of uninfected plants and cells of unadapted culture of the mycoplasma (6) and DNA (10 ng) extracted from cells of unadapted culture of the mycoplasma (7). The lane M corresponds to marker for length of DNA fragments (b.p.).

Changes in genome expression reorganization in bacteria adapting to UGC may explain the shift of bacterial metabolism and distortion or disappearance of virulence of some microorganisms[9,10,11,12]. However, this is the first report that shows that, at least in mycoplasma, adaptive processes were followed by an increase in pathogenicity. Further studies are needed to explore the exact mechanisms that permit adaptation to unfavorable growth conditions and changes in phytopathogenic potential.

## ACKNOWLEDGMENTS

The work was supported by the Russian Foundation for Basic Research (grant № 05-04-49435) and by the program of fundamental studies "Molecular and Cellular Biology" provided by the Russian Academy of Sciences, as well as by Government contract №02.442.11.7283.
